# Ankylosing Spondylitis and Rheumatoid Arthritis: Serum Levels of TNF-**α** and Its Soluble Receptors during the Course of Therapy with Etanercept and Infliximab

**DOI:** 10.1155/2014/675108

**Published:** 2014-03-24

**Authors:** Martin Schulz, Helmut Dotzlaw, Gunther Neeck

**Affiliations:** ^1^BIOMEDRO Ltd, Genetic Institute of the Justus-Liebig-University, Heinrich-Buff-Ring 58-62, 35392 Giessen, Germany; ^2^Rheumazentrum Prof. Dr. med. Gunther Neeck, Goethestraße 40, 18209 Bad Doberan, Germany

## Abstract

The effects of the TNF-**α** blockers infliximab or etanercept on the levels of TNF-**α**, TNF-receptor 1 (TNF-R1), and TNF-receptor 2 (TNF-R2), as well as the levels of the inflammation markers CRP and IL-6, were measured in ankylosing spondylitis (AS) and rheumatoid arthritis (RA) patients receiving treatment with either compound. We found that RA patients tend to have higher levels of TNF-**α** than both healthy individuals and AS patients prior to treatment (P < 0.05). We measured greatly increased levels of TNF-**α** in both the AS and RA etanercept patient groups during the course of treatment, while in the infliximab treated patients, the amount of TNF-**α** measured remained unchanged. Elevated TNF-**α** in the etanercept treated patients does not appear to be a significant risk factor for the spontaneous development of further autoimmune diseases in our study group. Increased levels of TNF-R1 were determined in both AS (P < 0.05) and RA (P < 0.001) patients when compared to healthy controls. In AS patients, the levels of TNF-R1 dropped significantly when treated with either infliximab (P < 0.01) or etanercept (P < 0.001). In contrast, the levels of this receptor remained unchanged in RA patients treated with either compound.

## 1. Background

Proinflammatory signaling that is activated by TNF-*α* is an important aspect in the pathology of rheumatic autoimmune diseases such as ankylosing spondylitis (AS) and rheumatoid arthritis (RA). TNF-*α* exerts its activity through binding to the membrane bound TNF-*α* receptors TNF-R1 and TNF-R2. In addition to their cell-bound forms, these receptors also exist as soluble molecules (sTNF-Rs) that are a result of enzymatic cleavage of the extracellular portions of the receptors [1]. These soluble receptors are free-floating in the serum and can bind to and act as natural TNF-*α* antagonists. It is thought that sTNF-R1 and sTNF-R2 modulate and balance the activity of TNF-*α* in the course of inflammatory events [2, 3].

Anti-TNF-*α* biologicals that complex TNF-*α* and neutralize its disease driving activities are beneficial drugs in the treatment of chronic inflammatory disorders. Infliximab, a humanized mouse monoclonal antibody against TNF-*α*, and etanercept, a soluble fusion protein comprising an epitope derived from the human TNF-receptor 2 fused to the Fc portion of human IgG, represent two different types of biotechnical engineered molecules developed for therapeutic targeting of TNF-*α* in inflammatory diseases. Both drugs are approved for the treatment of AS and RA as well as for other autoimmune diseases [4].

Although different autoimmune disorders share TNF-*α* as a major player in disease pathology, there are likely to be alterations in TNF-*α* regulation that can distinguish between different disease manifestations and respond to different types of TNF-*α* blocking strategies. Furthermore, studies have reported elevated levels of TNF-*α* in patients treated with TNF-*α* blockers and have speculated about the possible consequences thereof [5–8]. In order to see whether we could measure any differences in TNF-*α* regulation in patients with AS and RA being treated with infliximab and etanercept, we determined the serum concentration of TNF-*α* as well as the levels of the soluble TNF-receptors 1 and 2. The measurement of interleukin 6 and C-reactive protein in the serum samples was used to further assess the inflammatory activity in these patients. To follow the course of the disease, we calculated the Bath ankylosing spondylitis disease activity index (BASDAI) and the disease activity score 28 (DAS28) for the AS and RA patients, respectively, at the start of the study and after 12 weeks into treatment.

## 2. Methods

### 2.1. Study Participants

The study cohort consists of 45 healthy blood donors, 50 patients with ankylosing spondylitis (AS), and 48 patients with rheumatoid arthritis (RA). AS patients met the criteria of the European Spondyloarthropathy Study Group [9] and RA was diagnosed according to the classification criteria of the American College of Rheumatology [10]. None of the patients exhibited any comorbidities such as malignancies or psoriasis vulgaris, nor showed symptoms of any other chronic diseases. Patients that had been recently immunized, or had a history of viral infection such as HBV or HCV, were excluded from the study. All patients underwent anti-TNF-*α* therapy for the first time with either etanercept or infliximab. Twenty-two AS and 20 RA patients received etanercept 50 mg subcutaneously weekly during the study, and patients being treated with infliximab received IV infusions of 3 mg/kg body weight (28 RA patients) or 5 mg/kg body weight (28 AS patients) at 0, 2, and 6 and then every 8 weeks (RA patients) or 6 weeks (AS patients) into therapy. Patients treated with infliximab had been treated previously only with NSAIDs, whereas RA patients had received 2 or more DMARDs, including methotrexate, before infliximab or etanercept. RA patients treated with infliximab or etanercept received concomitant methotrexate combined with low-dose glucocorticoids with mean dosages of 15 mg/week and 5 mg/day, respectively. After obtaining written informed consent, serum samples were taken at different time points and stored at −80°C until assayed. Clinical and laboratory assessments were conducted prior to administration of the respective TNF-*α* blocker at baseline and at 2 and 12 weeks into therapy. The serum concentration of C-reactive protein (CRP) and the disease activity were determined. Assessment of the disease activity was performed before and after 12 weeks of therapy following the Bath ankylosing spondylitis disease activity index (BASDAI) for the AS patients and the disease activity score 28 (DAS28) was calculated for the RA patients.

The study was approved by the local ethics committee of the University of Rostock.

### 2.2. ELISA

Quantification of sTNF-R1, sTNF-R2, sTNF-*α*, and IL-6 in serum samples was performed using ELISA-kits (R&D Systems, Wiesbaden, Germany, Catalogue Numbers DY225, DY726, DY210, DY206, resp.) following the manufacturer's protocol. The detection limit was 10 pg/mL for all determinations.

### 2.3. Statistical Analysis and Graphical Representation

Statistical analysis was performed using GraphPad Prism software (San Diego, USA). One way ANOVA was used to compare parameters measured in the different study groups and repeated measures ANOVA were used to compare values obtained from the same patients at the three time points. ANOVA analyses were followed by Tukey's multiple comparison tests. Paired *t*-test was used to analyze changes in the disease activity scores during the study period. Comparisons among groups and among time points are depicted as box and whiskers plots. The boxes represent the range from the 25th to the 75th percentile and the whiskers represent the maximum and minimum values. The horizontal line through the box indicates the median.

## 3. Results and Discussion

Comparing the three groups analyzed in the present study, the age of the healthy controls and the AS patients is very similar with an age (mean ± SD) of 45 ± 12 and 45 ± 13 years in both groups. With a mean age of 56 ± 12 years in the RA group, these patients are significantly older (*P* < 0.001 for both comparisons). The gender distribution within the study cohort shows a higher proportion of females in the control group (25 of 45) and the RA patients (35 of 48), whereas females are underrepresented among the AS patients (21 of 50).

In the AS patient groups treated either with etanercept or infliximab, the anti-TNF-*α* therapy led to a reduction of the inflammatory activity as seen by a significant drop of the CRP concentration after 2 and 12 weeks into therapy (*P* < 0.01 for both treatments). The RA patients show a trend towards decreasing CRP levels during both treatments, but this effect does not reach statistical significance. The proinflammatory cytokine IL-6 also tends to decrease during therapy in all treatment groups, but only reached statistical significance in the AS patients treated with etanercept and infliximab at the 2 week time point (both *P* < 0.05). All serum parameters determined before and during anti-TNF-*α* therapy are listed in Table 1.

**Figure 1:**
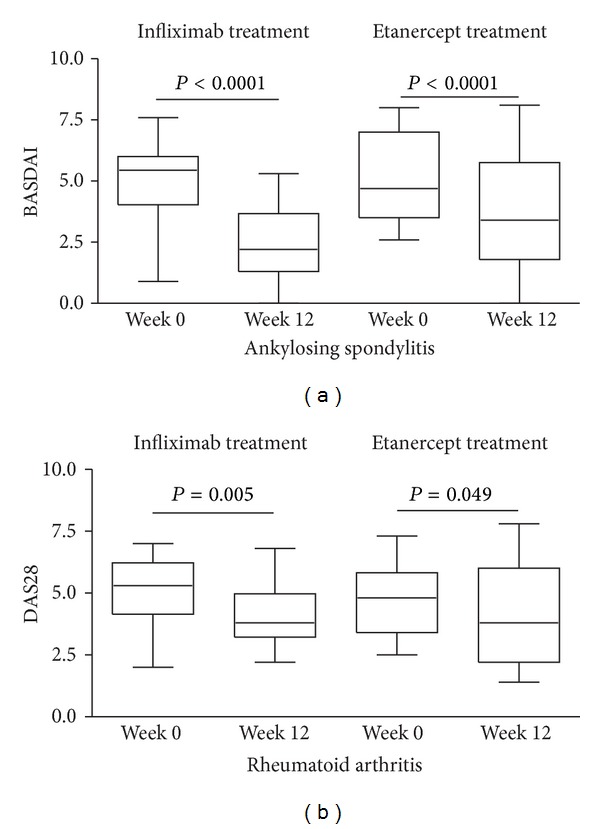
Changes in disease activity after 12 weeks of anti-TNF-*α* therapy. Disease activity scores were determined prior to the start of and at 12 weeks into treatment with infliximab and etanercept. Disease activity was assessed using the Bath ankylosing spondylitis disease activity index (BASDAI) and the disease activity score 28 (DAS28) in patients with ankylosing spondylitis (a) and rheumatoid arthritis (b), respectively. Significant differences in disease activity are indicated by the *P* values.

All four treatment groups showed a significant decrease in the disease activity scores (BASDAI for AS and DAS28 for RA) determined before the first administration of a TNF-*α* blocker and after 12 weeks into therapy (Figure 1). This response shows that 12 weeks of treatment with infliximab and etanercept leads to clinical improvement in both diseases in our study cohort.

### 3.1. Serum TNF-*α*

Prior to treatment, 32% (infliximab group) and 20% (etanercept group) of RA patients were found to be TNF-*α* positive (>10 pg/mL). This is around twice as many as TNF-*α* positive AS patients prior to treatment (14% and 9%, infliximab and etanercept groups, respectively, Table 1). The mean TNF-*α* level prior to treatment of 8.7 ± 17.9 pg/mL in RA patients was significantly higher (*P* < 0.05) than in AS patients (2.4 ± 7.2 pg/mL) and in healthy participants (2.5 ± 7.9 pg/mL).

**Figure 2:**
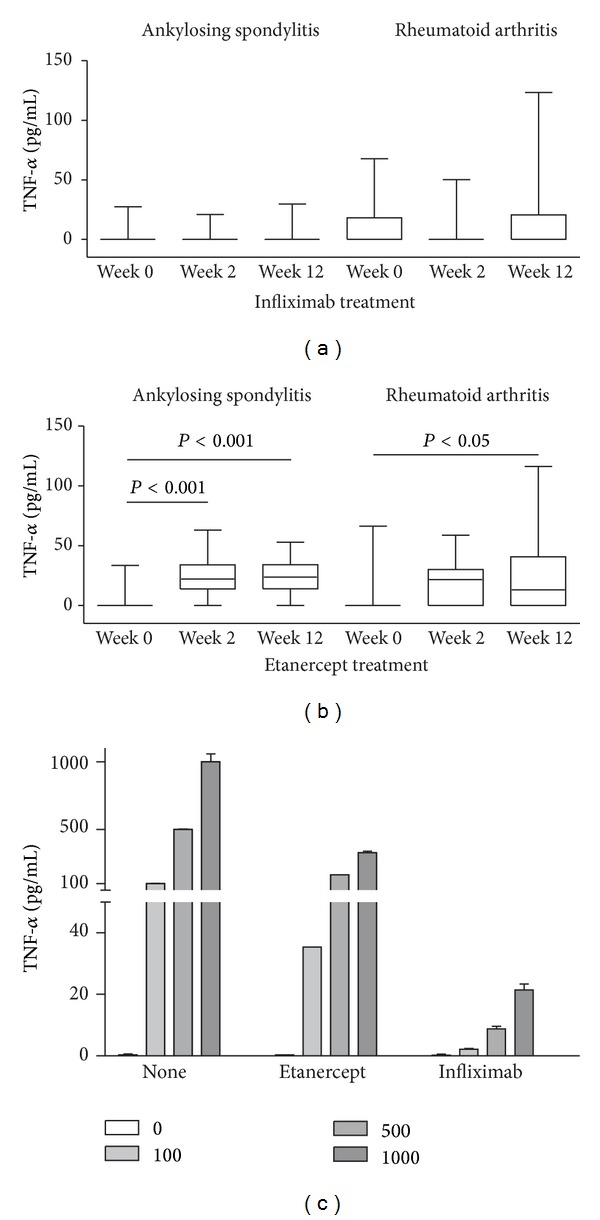
Influence of TNF-*α* blocking therapy on the serum TNF-*α* concentration in patients with ankylosing spondylitis and rheumatoid arthritis. Serum samples were taken at the indicated time points of therapy with infliximab (a) and etanercept (b). Significant changes in the TNF-*α* concentration during the course of therapy are indicated by the *P* values. To test for interference of TNF-*α* blockers, standard curves were performed using TNF-*α* ELISA in the presence of either 3 *μ*g/mL etanercept or 5 *μ*g/mL infliximab (c).

During the course of TNF-*α* blockade, no significant change in the level or in the percentage of TNF-*α* positive study subjects could be observed in the AS and the RA patients being treated with infliximab. Administration of etanercept led to a significant increase of the mean serum TNF-*α* concentrations in the AS patients (2.5 ± 8.3 to 23.4 ± 14.8 pg/mL, *P* < 0.001) as well as in the RA patients (5.8 ± 15.7 to 23.1 ± 28.7 pg/mL, *P* < 0.05) after 12 weeks into therapy (Figures 2(a) and 2(b)). This resulted in an increase in the number of TNF-*α* positive patients during etanercept treatment, with 96% and 82% of the AS patients, and 70% and 65% of the RA patients being TNF-*α* positive at the 2 week and 12 week time point, respectively. The amount of TNF-*α* detected in the presence of etanercept was not different when comparing the AS and RA patients during treatment (Table 1).

Our observation that the level of measured TNF-*α* does not increase in infliximab treated patients is in contrast to a previous study by Charles et al., in which a dose dependent increase in serum TNF-*α* in RA patients being treated with infliximab (cA2) was found [11]. We therefore tested whether the amount of TNF-*α* that is measured with the ELISA kits that we employed can be affected by either the presence of etanercept or infliximab in the sample. In order to do so, we mixed and incubated (30 minutes at room temperature) various concentrations of TNF-*α* with physiologically relevant amounts of either TNF-*α* blocker, and measured the amount of TNF-*α* using our anti-TNF-*α* ELISA assay. We found that both agents reduced the measured amount of TNF-*α*, with the effect by infliximab being much larger than that of etanercept (Figure 2(c)). As such, we are not able to interpret our measurements of TNF-*α* made in the presence of infliximab, since our assay system is not capable of measuring most of the TNF-*α* that is bound by this compound. Although we cannot rule out the possibility that autoantibodies to infliximab could also influence the quantitation of TNF-*α*, the fact that the presence of infliximab influences the amount of TNF-*α* measured in our assay might explain the different results that we obtained when compared with the study of Charles et al., that used a different ELISA assay system to measure TNF-*α* in the presence of infliximab.

In the past years, studies have emerged reporting increased serum concentrations of TNF-*α* as measured using ELISA assays in individuals being treated with etanercept for various conditions including rheumatic autoimmune disease [12]. The elevated serum levels could be explained by both an increased half-life of TNF-*α* when complexed with etanercept [13], and an upregulated expression and/or release of soluble TNF-*α*, that would be consistent with the observation that etanercept can enhance the capacity of immune cells to produce TNF-*α* [14], or possibly by the different biochemical and pharmacokinetic properties of the two anti-TNF-*α* agents.

While TNF-*α* is thought to be biologically inactive when complexed with the receptor fusion protein etanercept, the idea has been put forward that increased serum levels of TNF-*α* resulting from etanercept treatment can trigger autoimmune reactions such as psoriasis and Crohn's disease. The possible consequences thereof have been the subject of some debate, and it has been suggested that routine monitoring of serum TNF-*α* should be performed in all patients being treated with anti-TNF-*α* biologicals for this reason—even though the interpretation of such measurements remains unclear [5–8]. Although TNF-*α* is increased in the vast majority of the patients treated with etanercept in this study, we had not observed any new autoimmune symptoms such as psoriasis in these patients. Although we did measure differences in the levels of TNF-*α* in individual patients during the course of therapy with etanercept, the reasons for these differences are unclear, and we have no evidence to support the routine measurement of TNF-*α* in patients being treated with these compounds.

### 3.2. Soluble TNF-*α* Receptor 1 and 2

**Figure 3:**
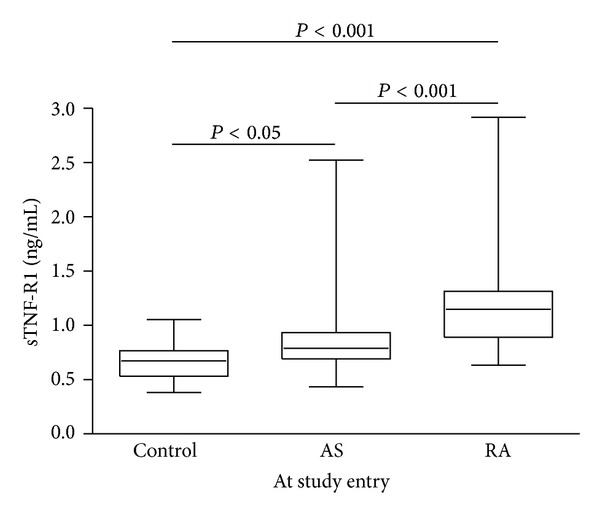
Comparison of serum TNF-R1 levels in healthy controls, AS, and RA patients. Patient serum samples were taken prior to the start of anti-TNF-*α* therapy. Significant differences in the receptor concentration found in the study groups are indicated by the *P* value.

Serum TNF-R1 showed elevated concentrations in both the AS and RA patient groups prior to anti-TNF-*α* treatment when compared to the controls (Figure 3). The mean concentration in the healthy blood donors was 0.66 ± 0.17 ng/mL compared to 0.89 ± 0.39 ng/mL in the AS patients and 1.22 ± 0.49 ng/mL in the RA patients. The differences among the groups reached statistical significance with *P* < 0.05 (AS versus control) and *P*< 0.001 (RA versus control and RA versus AS). No significant differences in the serum TNF-R2 concentration could be detected when comparing the healthy control subjects with the two patient groups at the 0 week time point. The mean concentrations were 2.86 ± 1.80 ng/mL in the healthy subjects and 2.79 ± 1.33 and 3.22 ± 1.49 ng/mL in the AS and RA patient group, respectively. These results are in partial agreement with previously published data on RA patients, which showed elevated levels of both TNF-*α* receptors when compared with healthy controls [15, 16].

After 2 weeks into therapy, the AS patients showed a significant decrease in TNF-R1 levels when treated with either infliximab or etanercept. This effect was maintained at the 12 week time point, resulting in a 15 and 23% reduction of the receptor concentrations as compared to the pretreatment measurement (*P* < 0.01 and *P* < 0.001 in the infliximab and the etanercept groups, resp.). In the RA patient groups, that displayed the highest initial receptor levels, neither of the TNF-*α* blockers used affected the serum TNF-R1 concentrations during the study period (Table 1).

The decrease in serum concentration of TNF-R1 in AS patients treated with infliximab and etanercept is in line with the idea that the level of soluble TNF-R1 is connected to the activity of TNF-*α* and inflammatory events as discussed in context of rheumatoid arthritis and other diseases in which TNF-*α* is involved [17–19]. Although we cannot exclude the possibility that the higher dosage of infliximab used in treating AS patients might at least partly affect the levels of TNF-R1 in this group, the fact that the levels of TNF-R1 are not affected by either TNF-*α* blocker in RA patients might be explained by a distinct regulation of TNF-*α* signaling in AS compared to RA patients. Our study suggests that soluble TNF-R1 might be a good marker of inflammation in AS patients. It is an easily measured parameter that could be accurately quantified in every patient, whereas TNF-*α*, CRP, and IL-6 are often below the detection limits in routinely used assays.

**Figure 4:**
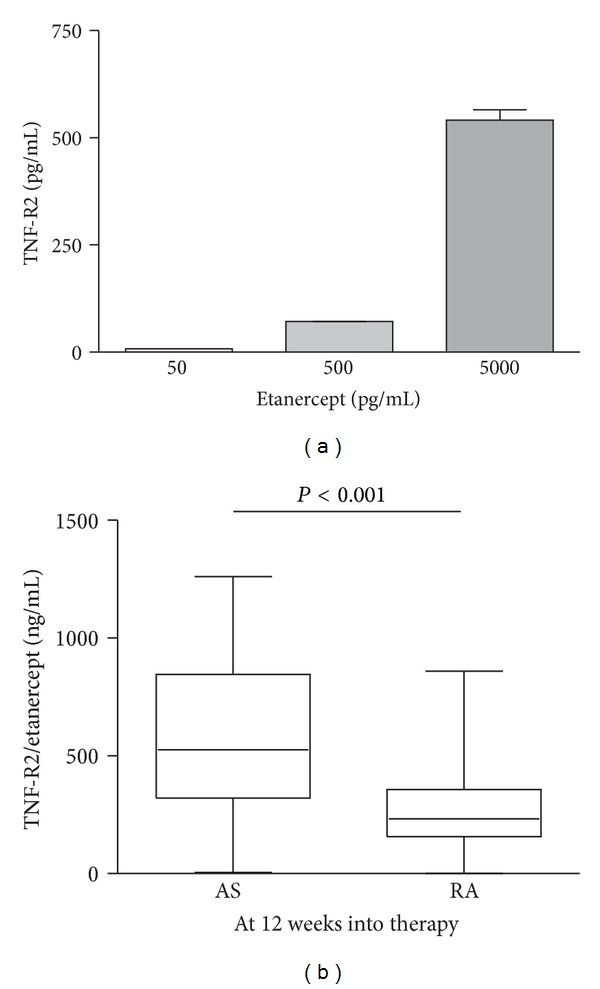
Detection of etanercept in the TNF-R2 ELISA. Serial dilutions of etanercept were assayed in the TNF-R2 ELISA and compared to recombinant standards (a). Patient sera at the 12 week time point of etanercept treatment were analyzed using TNF-R2 ELISA (b). The significant difference between the patient groups is indicated by the *P* value.

No significant change in TNF-R2 was observed during infliximab treatment of the AS and RA patients. In the presence of etanercept, the measurement of serum TNF-R2 was interfered with by the TNF-R2 portion of the drug, and this resulted in values that were out of the range of the assay. We analyzed serial dilutions of etanercept using our TNF-R2 ELISA and found that the reactivity of etanercept in this immunoassay was about 10 to 15% when compared to the recombinant TNF-R2 standard calibrators (Figure 4(a)). We therefore diluted etanercept treated patient sera 1000-fold and reassayed them in the TNF-R2 ELISA. The ELISA determinations were about 100-fold higher as compared to endogenous TNF-R2 levels prior to the start of treatment, with mean concentrations corresponding to 570 ± 348 and 280 ± 198 ng/mL in AS and RA patient sera, respectively. Although etanercept is only partially recognized in the TNF-R2 ELISA, the assay can still be used to compare the relative concentration of etanercept in the patient sera. At the 12 week time point, when an established level of etanercept can be expected, the concentrations detected as well as the variation of the levels found in the group of AS patients were significantly higher (*P* < 0.001) when compared to the RA patients (Figure 4(b)). To test the possibility that high levels of TNF-*α* might interfere in the TNF-R2 ELISA, we mixed etanercept with varying concentrations of TNF-*α* and assayed these samples using ELISA. We found no effect of TNF-*α* up to a concentration of 1000 pg/mL (data not shown).

As both AS and RA are treated by self-administration of etanercept, both at the same dosage and time interval, our measurements indicate that distinct differences in drug metabolism between our patient groups exist. This is in contrast to a study where no significant change in etanercept pharmacokinetics could be detected when comparing AS patients with RA patients [20]. It is unlikely that the difference in the composition of our patient groups might account for the different etanercept levels we observe between AS and RA patients, since it has been shown that etanercept disposition does not differ between males and females and does not vary with age in adult patients [21, 22]. The fact that two patients with AS and two patients with RA displayed TNF-R2 concentrations at the 12 week time points similar to those found prior to treatment begins could be explained by noncompliance in these patients. This could also explain why elevated levels of TNF-*α* were not detected in these patients. Interestingly, a recent study found that the levels of etanercept at 3 months into treatment can predict response to therapy at 6 months, suggesting that the monitoring of etanercept during treatment might be of prognostic value [23].

## 4. Conclusions

The increase in measurable TNF-*α* frequently observed upon etanercept treatment likely reflects biochemical and physiological properties in the regulation of TNF-*α* and was not identified as being a risk factor for triggering further inflammatory reactions in our study. In AS patients, the measurement of TNF-*α* receptor 1 might be useful as a biological indicator of TNF-*α* blocker activity. The determination of serum etanercept might be useful in the monitoring of medication compliance and to allow for a quantification of the steady state concentration of these compounds in individual patients.

## Figures and Tables

**Table 1:** Serum parameters in AS and RA patients treated with infliximab and etanercept, respectively.

Week	Ankylosing spondylitis						Rheumatoid arthritis					
	Infliximab			Etanercept			Infliximab			Etanercept		
	*N* = 28			*N* = 22			*N* = 28			*N* = 20		
	0	2	12	0	2	12	0	2	12	0	2	12
CRP mg/mL	18.4 ±21.2	**5.9** **±6.9**	**7.2** **±9.5**	11.24 ±15.5	**2.4** **±5.0**	**3.1** **±4.8**	20.9 ±27.5	7.7 ±14.9	13.0 ±24.6	26.5 ±31.2	19.4 ±23.3	21.8 ±28.4

IL-6 pg/mL	3.1 ±7.4	**0**	0.4 ±1.9	15.2 ±18.3	**3.8** **±8.7**	6.3 ±10.8	11.7 ±28.8	7.5 ±21.4	5.2 ±7.1	19.8 ±25.7	13.3 ±27.5	10.9 ±20.4

TNF-*α* pg/mL	2.3 ±6.3	1.6 ±4.9	2.7 ±7.7	2.5 ±8.3	**25.6** **±14.8**	**23.4** **±14.8**	10.7 ±19.3	6.0 ±13.0	13.0 ±27.2	5.8 ±15.7	**19.2** **±15.8**	**23.1** **±28.7**
Patients >10 pg/mL	4 14%	3 11%	3 11%	2 9%	**21** **96%**	**18** **82%**	9 32%	5 18%	9 32%	4 20%	**14** **70%**	**13** **65%**

TNF-R1 ng/mL	1.01 ±0.47	**0.86** **±0.30**	**0.86** **±0.27**	0.74 ±0.16	**0.55** **±0.16**	**0.57** **±0.17**	1.23 ±0.58	1.06 ±0.45	1.20 ±0.79	1.21 ±0.35	1.10 ±0.36	1.17 ±0.39

TNF-R2 ng/mL	3.08 ±1.09	2.84 ±0.95	3.10 ±1.06	2.43 ±1.55	474* **±319**	570* **±348**	4.03 ±1.33	3.60 ±1.38	3.95 ±1.20	2.02 ±0.66	278* **±144**	280* **±198**

The serum concentrations of CRP, IL-6, TNF-*α*, TNF-R1, and TNF-R2 are reported as mean values ± SD. Numbers in bold indicate significant differences in the concentration at 2 or 12 weeks into therapy, when compared to the pretreatment level; *indicates TNF-R2 determinations which were interfered with by etanercept.

## Abbreviations

ACR: American College of Rheumatology
AS: Ankylosing spondylitis
BASDAI: Bath ankylosing spondylitis disease activity index
CRP: C-reactive protein
DAS28: Disease activity score 28
IL-6: Interleukin-6
RA: Rheumatoid arthritis
sTNF-*α*: Soluble tumor necrosis factor *α*
sTNF-R1/2: Soluble tumor necrosis factor receptor 1/2.
